# Acute exercise improves emotion regulation in college students following social stress

**DOI:** 10.3389/fpsyg.2026.1808685

**Published:** 2026-04-20

**Authors:** Rui Luo, Lei Cui, Qiqi Shen, Xue Han, Dongling Wang, Xinnan Li

**Affiliations:** 1 College of P.E. and Sports, Beijing Normal University, Beijing, China; 2 Mental Health Education and Counseling Center, China University of Labor Relations, Beijing, China; 3 Department of Psychology, Roosevelt University, Chicago, IL, United States; 4 Peking University Health Science Center, Beijing, China; 5 Department of Physical Education, Beijing Technology and Business University, Beijing, China

**Keywords:** acute exercise, college student, depression, emotion regulation, social stress

## Abstract

**Objective:**

Despite findings that acute exercises can regulate emotions, more specific research is needed to distinguish more efficient exercise types. This paper examines the effects and differences of three acute moderate exercise types on emotion regulation after a social stress event.

**Methods:**

A total of 72 college students were randomly assigned into 4 groups: single aerobic group (Gsa), paired aerobic group (Gpa), dynamic stretching group (Gds), and sitting group (Gs) as a control group. Each participant in groups experienced a Trier Social Stress Test (TSST) for social stress induction and a 15-min group intervention. The participants’ states on emotions were measured 3 times by the Profile of Mood State (POMS), and their variations were statistically analyzed.

**Results:**

(1) The scores of Stress, POMS Tension and Vigour significantly reduced in all 4 groups. (2) The scores of POMS Anger significantly increased in both Gsa and Gpa, and the score of Total Mood Disturbance (TMD) significantly increased only in the Gpa. (3) The scores of POMS Depression, Confusion and TMD significantly reduced in the Gds, specifically in which POMS Depression reduced more than Gsa or Gpa, and TMD reduced more than Gpa.

**Conclusion:**

The three acute moderate exercise types exhibited no advantage on alleviating the stress and tension of college students after social stress event, whereas the acute dynamic stretching exercise shew better effects on regulating negative emotions, especially on depression than the aerobic exercise.

## Introduction

Social stress can lead to emotional issues which not only affect social functioning, but also have pathological consequences on human reproductive, immune, and metabolic functions if the stress is prolonged (Miczek, 2010; Parker et al., 2022). College life is stressful for many students, and social stress increases the prevalence of multiple mental disorders (Eirini et al., 2020). While there are several coping strategies, exercise is one of the most effective ways to regulate emotions (Shiota and Kalat, 2017). Studies have shown that exercise not only alleviates negative emotions, reduces anxiety (including both state anxiety and trait anxiety), lowers the risk of depression, and improves sleep (2018 Physical Activity Guidelines Advisory Committee, 2018; Lam and Riba, 2016; Guo and Mao, 2017; Noetel et al., 2024; Hamer et al., 2006), but also enhances positive emotions (Berger and Motl, 2000), increases feelings of pleasure, happiness, and satisfaction (Penedo and Dahn, 2005; Jiang and Chen, 2014). Although many college students have been used to relieve stress and regulate emotions through a bout of exercise, it is fascinating to regulate emotions quickly and effectively after experiencing social stress.

On quickness of emotion regulation, even as little as 10–15 min of aerobic exercise (AE) can improve mood (Ebert, 1977), although the benefits are more significant when you exceed 20 min (Cooper, 2020). Another study indicated that a moderate-intensity stationary cycling session lasting just 15 min can significantly benefit in mood and cognition for people of all ages (Hogan et al., 2013). Basso and Suzuki categorized acute exercise into three timeframes: less than 15 min, 16–45 min, and over 45 min (Basso and Suzuki, 2017). Within all three timeframes, studies have confirmed the mood-regulating effects of acute exercise on the brain and behaviors. However, Daley and Welch found no substantial difference in the positive effects on individuals’ moods between 15-min and 30-min exercise sessions, suggesting that a relatively brief 15-min exercise can suffice for mood regulation (Daley and Welch, 2004). Regarding exercise intensity, many studies have shown a curvilinear (inverted U-shaped) relationship between it and positive mood, with moderate-intensity exercise having more pronounced mood-enhancing benefits than light and vigorous exercise (Ligeza et al., 2021; Meyer et al., 2016, 2022). In short, studies have shown that engaging in a bout of acute exercise can have a positive effect on regulating emotion, and we make a grounded assumption that the critical effect duration and intensity is most likely 15 min and moderate-intensity.

If exercise is medicine, the type is the ingredient, an essential element for the effect of emotion regulation. Then we wonder that which type of acute moderate exercise has better effect on emotion regulation. A cross-sectional study involving over 1.2 million people has showed that individuals who engaged in popular team sports, cycling, aerobic and gym exercises had the lowest mental health burden (Chekroud et al., 2018). The result indicates that exercises to promote mental health exhibit difference in their effects, which may be attributed to different characteristic factors. A review of evidence-based research suggests that AE are associated with most mental health benefits and better feelings than AE (2018 Physical Activity Guidelines Advisory Committee, 2018). Besides, the mood-regulating effect of acute AE has been confirmed by many recent studies (Bernstein and McNally, 2017a, 2017b; Edwards et al., 2018; Gao et al., 2022). Firstly, aerobics can be identified as one of the effective factors that impacts the emotion regulation. Secondly, in addition to internal factors such as personality and motivation, psychosocial factors such as interpersonal interactions and social support during exercise also can have a significant impact on mental health promotion (Cox, 2011; Jiang et al., 2015). Thirdly, stretching has been shown to reduce both physiological and subjective muscle tension, thereby mitigating negative emotions and reducing stress hormones (National Academy of Sports Medicine, 2021; Fahmy, 2021). These current studies have shown the emotion regulation potency of aerobics, interpersonal action and stretching, so there is still a need for studies to distinguish the acute effects of various types of exercise on emotion regulation.

The purpose of this study was to investigate which type of acute exercise is more effective in regulating emotions in college students following a social stress event. It was hypothesized that, under conditions of matched exercise duration and intensity, the emotion regulation effects would differ among exercise types incorporating different characteristic factors. Three characteristic factors (aerobics, interpersonal interaction and stretching) were selected to formulate three sets of 15-min moderate-intensity exercise intervention programs: single AE, paired AE, and dynamic stretching exercise. A control set (silent sitting) was added to compare as a baseline. The experimental method was applied to assess the effects of these interventions on emotion regulation, which aims to find out better exercise type(s) for regulating stressful emotions, and for the design of larger follow-up studies and eventually the development of educational concepts and exercise prescription.

**Figure 1:**
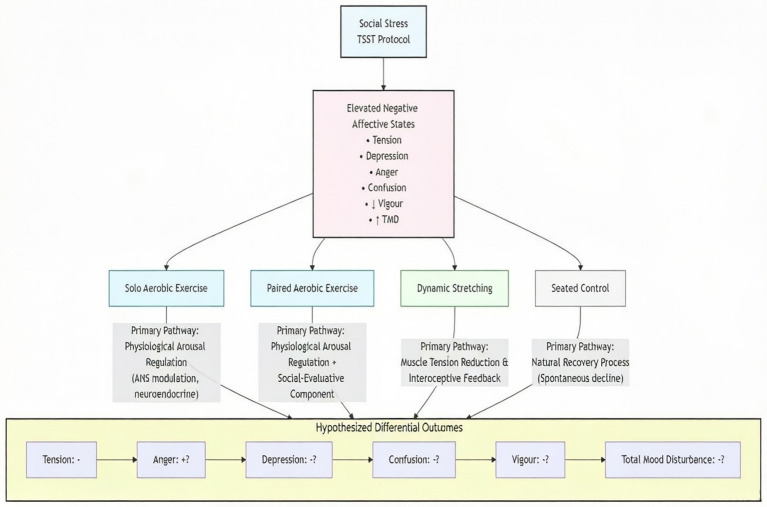
Mechanistic model of the effects of acute exercise on emotion regulation after social stress.

Based on the existing literature, we propose a conceptual model (see Figure 1) to delineate the hypothesized pathways linking acute exercise to emotion regulation following social stress. Specifically, social stress triggers negative affective states (e.g., tension, depression, confusion). Different types of acute exercise are posited to regulate these emotions through distinct primary mechanisms. Aerobic exercise (both solo and paired) primarily modulates emotional arousal via physiological pathways (e.g., alteration of autonomic nervous system activity and neuroendocrine responses), whereas dynamic stretching targets emotion regulation by reducing physiological and subjective muscle tension, which is closely linked to states of depression and anxiety. Furthermore, the interpersonal interaction inherent in paired exercise may introduce an additional layer of cognitive appraisal (e.g., social-evaluative concern or performance pressure), which could paradoxically counteract the stress-reducing benefits of physical exertion, particularly for negative emotions like anger.

The primary purpose of this study was to empirically compare the immediate effects of three distinct types of 15-min moderate-intensity acute exercise on emotion regulation in college students following a standardized social stressor. Informed by the conceptual model, we formulated the following specific hypotheses:

*H1*: Compared to a seated control condition, all three acute exercise interventions (solo aerobic, paired aerobic, and dynamic stretching) will lead to a greater reduction in overall mood disturbance (Total Mood Disturbance score) following the social stress task.

*H2*: Dynamic stretching will be more effective than both aerobic exercise modalities (solo and paired) in reducing scores on the Depression and Tension subscales of the Profile of Mood States, due to its targeted effect on relieving muscular tension.

*H3*: Paired aerobic exercise will be less effective than solo aerobic exercise and dynamic stretching in reducing scores on the Anger and Confusion subscales, as the interpersonal component may introduce additional cognitive or social stress. (As shown in Figure 1)

## Method

### Data collection and ethical statements

Data collection and ethical considerations were conducted in strict accordance with the guidelines established by the Human Subjects Research Ethics Review Committee of Beijing Normal University. This study has been approved by the Human Subjects Research Ethics Review Committee of Beijing Normal University (ref: # IRB_B_0015_2022001). Participants were recruited from 01 November 2021 to 30 November 2022. All the participants were recruited volunteers serving for an international event (to protect their privacy, the information are not mentioned in this article). Before the volunteer interview, they were informed and consented to take a comprehensive competency assessment and the results would be used in research. Throughout the study, the privacy, accuracy and integrity of the data were strictly protected.

### Participants

The 72 participants (14 male and 58 female) were college student volunteers from a Chinese university, Han nationality and aged 20.13 ± 0.44 years (mean±s). They were recruited for an international event service as volunteers. Given that our sample consisted predominantly of female participants, the primary findings of this study reflect the effects of acute exercise on emotion regulation following social stress within a female undergraduate population. Future research with gender-balanced samples is needed to examine the generalizability of these effects and to investigate potential gender differences in exercise-emotion responses.

### Research design

The study used a 4 (group) ×3 (measurement time) two-factor mixed quasi-experimental design. All 72 students were randomly assigned into four groups of 18 participants: a single aerobic group (Gsa), a paired aerobic group (Gpa), a dynamic stretching group (Gds), and a sitting group (Gs). Each group differed only in exercise intervention protocol. The experimental procedures, stress tasks, and dependent variable measurements were all kept the same and performed sequentially in 1 day in order.

The experimental procedure for each group consisted of the following five steps: (1) Baseline measurement: participants entered the preparation room in advance, they were asked to scan the QR code with their cell phones to complete an online questionnaire 15 min before the stress task. (2) Implementation of the stress task: the Trier Social Stress Test (Kirschbaum et al., 1993) was used to elicit participants’ stress responses. The test lasted 20 min and consisted of a 10-min preparation session and a 10-min testing session. (3) Post-stress task measurement: after completing the stress task in the test room, participants were asked to scan the QR code again to complete the questionnaire. (4) Exercise intervention or sitting: participants were asked to go to the exercise hall after completing the questionnaire. Exercise intervention or sitting was implemented after all participants arrived. (5) Measurement after intervention or sitting: after the intervention or sitting, participants were asked to scan the QR code again to complete the questionnaire.

**Figure 2:**
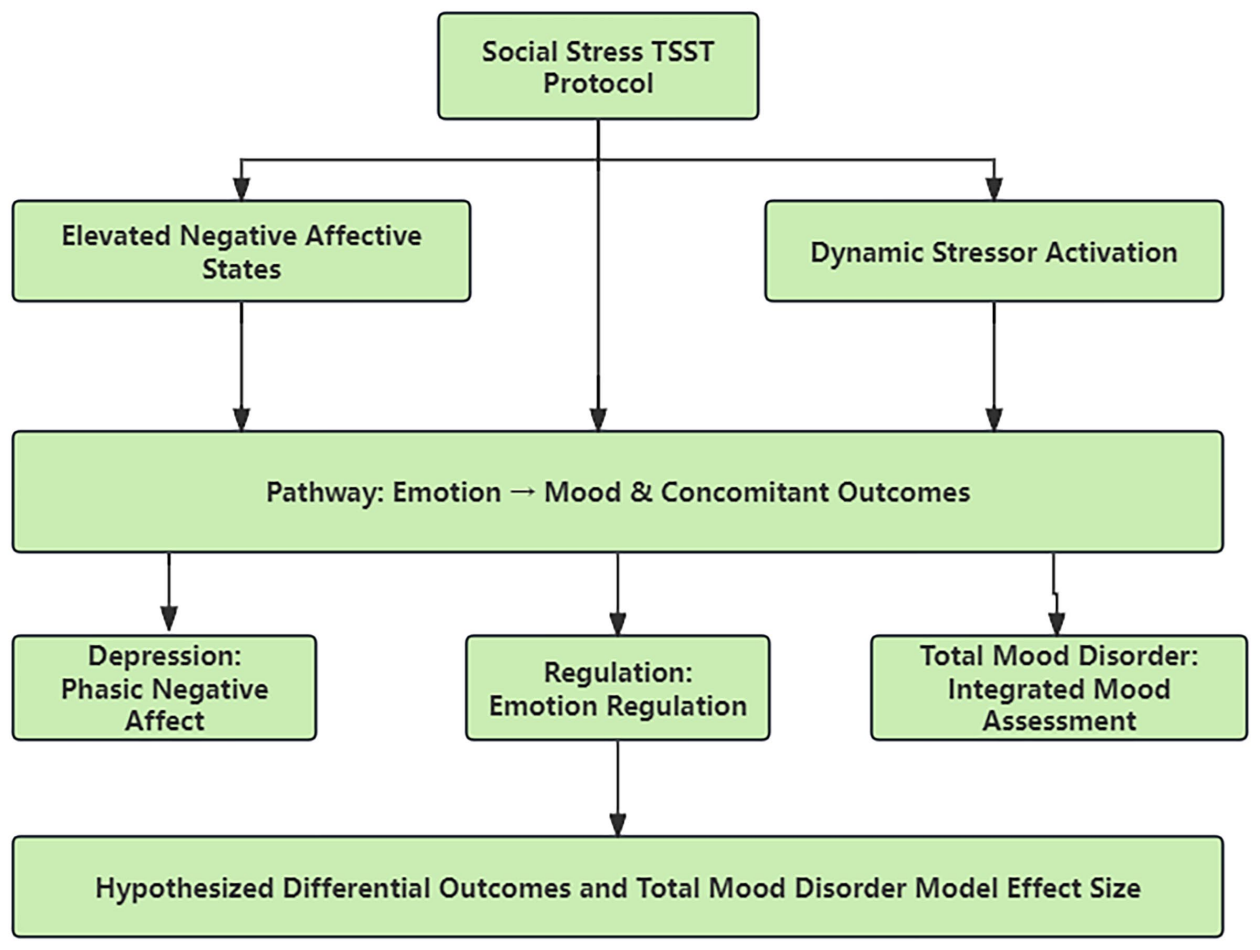
Experimental flowchart of the TSST stress task and subsequent exercise intervention.

Researchers supervised the whole execution of research design with the administrators of this volunteer project. All the interviewers of TSST participated in two sessions of TSST’s operation training. They were divided into 18 + 1 interview panels, a panel served as a backup. The operation was practiced by each interview panel and supervised to meet the standard by researchers. To ensure a unified intervention for all participants in one intervention group, we conducted TSST at the same time in different interview rooms as shown in Figure 2.

### Instruments

*Stress-inducing instrument*: The trier social stress test (TSST) was employed as the stress-inducing instrument. The TSST is a standardized, widely used protocol for reliably inducing moderate psychosocial stress in a laboratory setting, primarily through a simulated public speaking task combined with mental arithmetic. It is a widely recognized international assessment for inducing social stress and has demonstrated good applicability among Chinese participants (Yang et al., 2011). The procedure for eliciting stress in the present study was as follows: The procedure for eliciting stress was as follows: Participants underwent a stress-inducing job interview simulation after baseline measurements. They were given a test paper with instructions for a 10-min preparation period, followed by a 10-min interview divided into two parts. The first part required answering prepared questions, while the second involved a 5-min mental math challenge starting from 1,022 and subtracting 13. The examiner provided time cues and corrections for errors, concluding the interview after the allotted time or upon completion of the math task.

*Measuring scales*: Participants’ emotions were measured by the Profile of Mood States (POMS) in Chinese version (Zhu, 1995), a 5-point scale comprising 40 items in 7 subscales: “Tension”, “Anger”, “Fatigue”, “Depression”, “Vigour”, “Confusion”, and “Esteem-Related Affect”. Total Mood Disorder Score (TMD) can be calculated by summing the totals for the negative subscales and then subtracting the totals for the positive subscale: TMD = [Tension + Depression + Anger + Fatigue + Confusion] – [Vigour + Esteem-Related Affect]. In addition, one question about stress was added with a 5-point scale, “Please choose your level of stress at this moment: very stressful; stressful; between stressful and relaxed; relaxed; very relaxed.”

### Exercise intervention programs

Frequency, intensity, time, and type (FITT) of exercise are the critical factors in designing the exercise intervention program. For the research objective, the exercise frequency was set at once per session, in which time lasted for 15 min. Exercise intensity aimed for a moderate intensity according to the ACSM’s guideline, and was monitored by a sports watch to maintain a heart rate of 64%–76% of maximum heartrate (220-age) (American College of Sports Medicine, 2017). In terms of exercise types, we incorporated three characteristic factors: aerobics, interpersonal interaction and stretching, which are known to have a strong correlation with emotion regulation. Three distinct 15-min exercise intervention programs were developed to represent each of the three factors, and silent sitting was included as control condition. The exercise activities were leaded by an experienced physical educator who was familiar with all 3 programs. And the activities’ demonstration was also played on a 3.78 m*5.86 m screen in front of the participants. Participants in all exercise groups (Gsa, Gpa, Gds) wore Polar sports watches for continuous heart rate monitoring. Researchers monitored the heart rate in real-time to ensure it remained within the target range (64%–76% of HRmax). Standardized verbal prompts (e.g., “Please increase your movement amplitude”) were given if a participant’s heart rate fell below the target. To minimize interpersonal interaction as a confounding variable, researchers maintained neutral and minimal verbal contact with all participants throughout the session, delivering only standardized instructions.

This protocol of real-time monitoring with immediate standardized prompting ensured that exercise intensity was accurately maintained at the prescribed moderate level for all participants in the active intervention groups. Such researcher-supervised heart rate control is a well-established methodological practice in exercise psychology research to ensure fidelity to the intended exercise intensity prescription, thereby isolating the effects of exercise type from those of exercise intensity (American College of Sports Medicine, 2017).

Further details of these programs can be found in Table 1.

**Table 1:** Acute exercise intervention programs list.

Groups	Exercise intensity	Exercise time	Exercise types	Specific exercise activities
Gsa	Moderate	15 min	Single aerobics	Wide diagonal uppercut; alternating hip kicks; side-turn punch; quick-step punch; four-point punch; stepping and raising arms.
Gpa	Moderate	15 min	Paired aerobics	Paired activities: deep squat jumps; trot; alternating lunge; deep squat catching high leg raise; alternating leg raise; dynamic swallow balance.
Gds	Moderate	15 min	Dynamic stretching	Calf stretch; front thigh stretch; back stretch; back thigh stretch; shoulder stretch; greatest stretch.
Gs	N/A	15 min	Sitting	No exercise, sitting quietly in a chair.

### Statistics

The data were collected and organized on the Questionnaire Star platform, and statistical analysis was conducted by SPSS25.0. On the experiment day, two participants were unable to complete the experimental task due to health issues, consequently, their data were excluded from the result analysis. Given the relatively small sample sizes in this study, non-parametric statistical methods were employed. Specifically, the Paired Samples Wilcoxon Signed Rank Test was employed to assess differences in dependent variables between the pre-test and post-test within each group. The Kruskal–Wallis *H* Test was employed to test variations in the change of dependent variables among the groups before and after the intervention. Statistical significance was set at *p* < 0.05.

## Results

### Effects of social stress induction

To examine the stress-inducing effects of TSST, the Paired Samples Wilcoxon Signed Rank Test was employed to compare the stress scores’ difference between pre and post TSST in each group. The statistic results in Table 2 revealed that the stress significantly increased in all groups after TSST (*p* < 0.05). These findings also indicated that TSST could effectively induce stress.

**Table 2:** Stress Scores pre and post TSST in each group and test statistics.

Groups	Stress pre-TSST Mdn (Min-Max)	Stress post-TSST Mdn (Min-Max)	*Z*	*p* value
Gsa (*n* = 16)	2.5 (2–4)	3 (2–5)	−2.84	0.005*
Gpa (*n* = 18)	3 (2–5)	3 (2–4)	−2.18	0.029*
Gds (*n* = 18)	3 (1–4)	3 (1–4)	−2.50	0.013*
Gs (*n* = 18)	2.5 (1–4)	3 (2–4)	−2.50	0.012*

*Indicates *p* < 0.05.

### Homogeneity of the intervention samples after stress induction

The Kruskal–Wallis *H* Test was employed to assess the homogeneity of the intervention samples after stress induction. The results showed no significant differences (*p* < 0.05) among the groups after TSST in Table 3. In other words, the dependent variables exhibited samples’ homogeneity before the interventions.

**Table 3:** Scores on dependent variables and test statistics after TSST.

Dependent variables	GsaGsa Mdn (Min-Max)	Gpa Mdn (Min-Max)	GdsGds Mdn (Min-Max)	Gs Mdn (Min-Max)	*H*	*p* value
Stress	3 (2–5)	3 (2–4)	3 (1–4)	3 (2–4)	1.62	0.655
Tension	8.5 (6–15)	8 (6–16)	9 (6–17)	8 (6–15)	1.14	0.767
Anger	8.5 (7–19)	8 (7–13)	10 (7–21)	9 (7–18)	7.08	0.070
Fatigue	5.5 (5–15)	5 (5–7)	6.18 (5–14)	5 (5–14)	6.79	0.079
Depression	17 (6–26)	13.5 (7–23)	15 (7–21)	16.5 (6–22)	0.80	0.849
Vigour	12 (7–15)	10.5 (8–19)	11.89 (8–18)	12 (8–15)	2.33	0.507
Confusion	12.5 (7–17)	10.5 (5–14)	11 (6–16)	11 (5–14)	6.47	0.091
Esteem-related affect	9.5 (7–13)	9 (6–18)	9.91 (6–14)	9.5 (7–14)	1.82	0.611
TMD	131.5 (118–146)	125 (117–140)	130.13 (120–151)	128.5 (118–149)	7.57	0.056

### Effects of experimental interventions

The Paired Samples Wilcoxon Signed Rank Test was employed to examine the intervention effects of each group. The results showed in Table 4 as following: (1) Gsa experienced significant decrease in Stress, POMS Tension, Vigour and Confusion (*p* < 0.05), and significant increase in POMS Anger (*p* < 0.05) after the intervention. (2) Gpa experienced significant decrease in Stress, POMS Tension and Vigour (*p* < 0.05), and significant increase in TMD (*p* < 0.05) after the intervention. (3) Gds experienced significant decrease in Stress, POMS Depression, Vigour, Confusion and TMD (*p* < 0.05) after the intervention. (4) Gs had significant (*p* < 0.05) reductions in Stress, POMS Tension, Vigour and Esteem-Related Affect after sitting.

**Table 4:** Scores on dependent variables pre and post intervention and test statistics.

Dependent variables	Pre-intervention Mdn (Min-Max)				Post-intervention Mdn (Min-Max)				Intra-group *Z*				Inter-group *H*
	Gsa	Gpa	Gds	Gs	Gsa	Gpa	Gds	Gs	Gsa	Gpa	Gds	Gs	
Stress	3 (2–5)	3 (2–4)	3 (1–4)	3 (2–4)	2 (1–4)	2 (1–4)	2 (1–4)	2 (1–3)	−2.97*	−3.25*	−2.86*	−3.48*	1.22
Tension	8.5 (6–15)	8 (6–16)	9 (6–17)	8 (6–15)	6 (6–8)	6 (6–8)	7.05 (6–16)	6 (6–14)	−3.12*	−3.57*	−2.46*	−2.91*	3.74
Anger	8.5 (7–19)	8 (7–13)	10 (7–21)	9 (7–18)	11.43 (7–22)	10.5 (7–19)	12.5 (7–20)	9 (7–19)	−2.42*	−3.31*	−1.54	−0.88	6.45
Fatigue	5.5 (5–15)	5 (5–7)	6.18 (5–14)	5 (5–14)	5.5 (5–11)	5 (5–10)	6 (5–18)	5.5 (5–10)	−0.30	−0.51	−0.08	−0.09	0.69
Depression	17 (6–26)	13.5 (7–23)	15 (7–21)	16.5 (6–22)	15.5 (6–26)	13.3 (6–26)	11 (6–16)	14 (6–22)	−0.50	−0.75	−3.63*	−1.64	14.54*
Vigour	12 (7–15)	10.5 (8–19)	11.89 (8–18)	12 (8–15)	10.26 (6–16)	10 (8–15)	10.63 (6–16)	10 (6–14)	−2.55*	−2.14*	−2.71*	−2.69*	2.77
Confusion	12.5 (7–17)	10.5 (5–14)	11 (6–16)	11 (5–14)	10.57 (5–15)	9.57 (6–17)	10 (5–14)	11 (5–14)	−2.42*	−1.34	−3.28*	−1.12	13.68*
Esteem-related affect	9.5 (7–13)	9 (6–18)	9.91 (6–14)	9.5 (7–14)	9 (7–13)	8 (7–12)	9 (7–16)	9 (8–12)	−1.51	−1.66	−0.28	−2.12*	2.59
TMD	131.5 (118–146)	125 (117–140)	130.13 (120–151)	128.5 (118–149)	130.5 (118–146)	129.33 (118–146)	129.33 (116–146)	127 (114–146)	−0.10	−3.07*	−2.27*	−0.21	12.72*

Mdn, Median; Min, Minimum; Max, Maximum; Intra-Group *Z* is the statistical magnitude of the paired samples Wilcoxon signed rank test; inter-group *H* is the statistical magnitude of the Kruskal–Wallis H test; *indicates *p* < 0.05.

To examine whether there was a significant difference in variation (pre-intervention score minus post-intervention score) of dependent variables among 4 groups, the Kruskal–Wallis *H* Test was employed. The results in Table 4 showed that there was significant difference among groups (*p* < 0.05) in variation of POMS Depression, Confusion, and TMD scores. Post-hoc tests were used to compare all pairs of groups on the 3 variables.

*Between-group differences in POMS depression reduction*: according to the Bonferroni-adjusted *pb* values, the results showed that the difference between the Gds (*Mdn* = 3.87) and Gpa (*Mdn* = 0.5), the Gds (*Mdn* = 3.87) and Gsa (*Mdn* = 0.5) were significant (*pb* < 0.05), and none of the other comparisons was significant after Bonferroni adjustment. It indicates that dynamic stretching better regulates depression compared to single aerobic and paired AE.

*Between-group differences in POMS confusion reduction*: according to the Bonferroni-adjusted *pb* values, the results showed that the difference between the Gds (*Mdn* = 1.5) and the Gpa (*Mdn* = −0.5), the Gsa (*Mdn* = 1) and Gpa (*Mdn* = −0.5) were significant (*pb* < 0.05), and none of the other comparisons was significant after Bonferroni adjustment. It indicates that dynamic stretching and single AE both better regulates the confusion than paired AE.

*Between-group differences in TMD reduction*: according to the Bonferroni-adjusted *pb* values, the results showed that the difference between the Gds (*Mdn* = 5) and the Gpa (*Mdn* = −3) was significant (*pb* < 0.05), and none of the other comparisons was significant after Bonferroni adjustment. It indicates that dynamic stretching better reduces the level of mood disturbance compared to paired AE.

## Discussion

The results of our study not only confirm significant effects of the TSST on inducing stress, but also on different effects of 3 types of acute moderate exercise and the control condition (sitting). After a 15-min moderate-intensity exercise intervention of single AE, paired AE, dynamic stretching or sitting, all 4 groups occurred significant reduction of the Stress, POMS Tension and Vigour, yet there’s no inter-group difference on those variables. On the one hand, several previous studies with multiple-time data have indicated that participants in TSST would experience a declining process of stress and relative emotions of POMS after the test (Emma et al., 2010; Yang et al., 2011; Chen and Kong, 2016). Therefore, the reduction of POMS Tension and Vigour in exercise groups probably corresponds to the naturalistic course after the TSST. On the other hand, the results maybe reflect that the dosage of exercise is not enough to produce significant difference inter-group. This suggests that the 15-min moderate-intensity exercise intervention did not yield a better stress relief effect than sitting following a social stress event. Both short burst of exercise and sitting were effective in alleviating participants’ stress and tension. This outcome may correspond to the brain’s self-protective mechanism, which has been observed to activate restorative rest as a response to acute social stress. This restorative rest helps the body cope with elevated stress and relevant hormones, ultimately mitigating tension and anxiety (Yu et al., 2022). According to our experimental results, the 15-min moderate-intensity exercise intervention probably failed to produce a significant impact on this self-protective mechanism, and did not produce a superior emotion regulation effect that distinguished it from sitting. However, it’s noteworthy that even when participants were sitting silently, they still reported vigour declining due to coping with stress.

Contrastively, both 15-min moderate-intensity single and paired AE significantly elicited POMS Anger among participants, whereas dynamic stretching and sitting did not. The result is inconsistent with the results of previous studies (Bernstein and McNally, 2017a, 2017b), which may be related to the special exercise experience of acute AE. According to the physiological models to understand exercise fatigue and the adaptations (Noakes, 2000), at the beginning of acute AE, there is a process of internal adaptations (e.g., energy supply and muscle recruitment) for rapid transition into the exercise state, leading to the subjective experience of discomfort and reluctance to continue exercising, ultimately inducing the emotion of anger.

According to the experimental results, 15-min moderate-intensity dynamic stretching exercise better moderated depression mood than single or paired AE, and better reduced the TMD score than paired AE. Sudo & Ando’s study found that just 10 min of stretching incorporating yoga techniques and poses can significantly improve the mood state of physically inactive young adults, including reduction in Tension, Depression, Anger, Fatigue, and Confusion scores, but increase in Vigour score on POMS (Sudo and Ando, 2020). Another study also showed that 10 min of stretching exercise before bedtime among middle-aged women led to a reduction in menopausal and depressive symptoms (Kai et al., 2016). The consistent conclusions on emotion regulation of acute stretching exercise may be attributed to the mechanisms that stretching can reduce both physiological (myoelectric) and subjective muscle tension, enhance local blood circulation, and decrease stress hormones (National Academy of Sports Medicine, 2021). It’s worth noting that this study proved the advantages of acute stretching over acute AE with experimental evidence. For the efficacy of emotion regulation, especially depression alleviation, dynamic stretching emerges as a rapid and effective exercise type.

### Limitations

While the study contributes valuable insight into the effects of 3 types of acute exercise on emotion regulation after social stress event, it also exhibits several limitations in the following aspects. First, the sample in this experiment was a cluster sampling. Although random grouping was adopted, the small sample size large percentage of female participants and totally incorporated Chinese participants affected the representativeness of the experimental results. Second, there might be possible confounders on participant-characteristics which were not controlled subtly, such as fitness levels and social-related personalities. Third, stress and relevant emotions were only measured with one scale, and their physiological indicators data were missing because of technical fault, which lacked comprehensive verification of dependent variables. At last, due to the limited size of the experiment, the interaction effect of different characteristic factors on emotion regulation could not be determined. In future studies, larger sample and more dimensions of dependent variables’ measurement can be used to improve the explanatory power of different characteristic factors in exercise on emotion regulation. Based on present studies, the emotional regulation mechanism and dosage effect of specific exercise types (especially stretching exercise) with different frequency, intensity and duration can be further explored.

## Conclusion

After a social stress event, the three 15-min moderate exercise types have no advantage on alleviating the POMS Stress and Tension of college students. In addition, the acute AE is very likely to induce feeling of anger, and interpersonal interaction of paired exercise may not be recommended. It’s worth noting that the acute dynamic stretching exercise emerges as a better and more efficient type for negative emotion regulation.

## Data Availability

The original contributions presented in the study are included in the article/supplementary material, further inquiries can be directed to the corresponding author.

## References

[ref32] 2018 Physical Activity Guidelines Advisory Committee (2018). 2018 Physical Activity Guidelines Advisory Committee Scientific Report. Washington: U.S. Department of Health and Human Services (p. 779).

[ref1] American College of Sports Medicine (2017). ACSM’S Guidelines for Exercise Testing and Prescription. Philadelphia: Lippincott Williams & Wilkins.

[ref2] Basso J. C. Suzuki W. A. (2017). The effects of acute exercise on mood, cognition, neurophysiology, and neurochemical pathways: a review. Brain Plasticity 2, 127–152. doi: 10.3233/BPL-160040, 29765853 PMC5928534

[ref3] Berger B. G. Motl R. W. (2000). Exercise and mood: a selective review and synthesis of research employing the profile of mood states. J. Appl. Sport Psychol. 12, 69–92. doi: 10.1080/10413200008404214

[ref4] Bernstein E. E. McNally R. J. (2017a). Acute aerobic exercise hastens emotional recovery from a subsequent stressor. Health Psychol. 36, 560–567. doi: 10.1037/hea0000482 28277695

[ref5] Bernstein E. E. McNally R. J. (2017b). Acute aerobic exercise helps overcome emotion regulation deficits. Cogn. Emot. 31, 834–843. doi: 10.1080/02699931.2016.1168284, 27043051

[ref6] Chekroud S. R. Gueorguieva R. Zheutlin A. B. Paulus M. Krumholz H. M. Krystal J. H. . (2018). Association between physical exercise and mental health in 1·2 million individuals in the USA between 2011 and 2015: a cross-sectional study. Lancet Psychiatry 5, 739–746. doi: 10.1016/S2215-0366(18)30227-X, 30099000

[ref7] Chen G. Kong Y. (2016). Impact of Trier social stress test on children salivary cortisol secretion. Psychol. Dev. Educ. 32, 532–538. doi: 10.16187/j.cnki.issn1001-4918.2016.05.03

[ref8] Cooper S. L. (2020). Promoting physical activity for mental well-being. ACSMs Health Fit J 24, 12–16. doi: 10.1249/FIT.0000000000000569

[ref9] Cox R. H. (2011). Sport Psychology. Columbus: McGraw Hill.

[ref10] Daley A. J. Welch A. (2004). The effects of 15 min and 30 min of exercise on affective responses both during and after exercise. J. Sports Sci. 22, 621–628. doi: 10.1080/02640410310001655778, 15370492

[ref11] Ebert M. J. (1977). Type A and Type B Female’s Response to Acute Exercise: The Effect of Stress Reduction. Master’s thesis. Manhattan: Kansas State University.

[ref12] Edwards M. K. Rhodes R. E. Mann J. R. Loprinzi P. D. (2018). Effects of acute aerobic exercise or meditation on emotional regulation. Physiol. Behav. 186, 16–24. doi: 10.1016/j.physbeh.2017.12.037, 29309746

[ref13] Eirini K. Pim C. Yesica A. Jordi A. Randy P A. Jason B. . (2020). Sources of stress and their associations with mental disorders among college students: results of the World Health Organization world mental health surveys international college student initiative. Front. Psychol. 11:1759. doi: 10.3389/fpsyg.2020.01759, 32849042 PMC7406671

[ref14] Emma C. Nicholas T Van D. Harriet de W. (2010). Effects of acute progesterone administration upon responses to acute psychosocial stress in men. Exp. Clin. Psychopharmacol. 18, 78–86. doi: 10.1037/a0018060, 20158297 PMC4351805

[ref15] Fahmy R. (2021). Essentials of Corrective Exercise Training. Burlington: Jones & Bartlett Learning.

[ref16] Gao S. Wang X. Zhang L. Liu J. (2022). Selective effects of acute aerobic exercise on the mood of college students. Chin. J. Sports Med. 41, 459–464. doi: 10.16038/j.1000-6710.2022.06.005

[ref17] Guo L. Mao Z. (2017). Psychological benefits of physical activity or physical exercise. Adv. Psychol. 7, 1407–1418. doi: 10.12677/AP.2017.712174

[ref18] Hamer M. Taylor A. Steptoe A. (2006). The effect of acute aerobic exercise on stress related blood pressure responses: a systematic review and meta-analysis. Biol. Psychol. 71, 183–190. doi: 10.1016/j.biopsycho.2005.04.004, 15979232

[ref19] Hogan C. L. Mata J. Carstensen L. L. (2013). Exercise holds immediate benefits for affect and cognition in younger and older adults. Psychol. Aging 28, 587–594. doi: 10.1037/a0032634, 23795769 PMC3768113

[ref20] Jiang C. Chen T. (2014). The effect of physical activity on affect. Adv. Psychol. Sci. 22, 1889–1898. doi: 10.3724/sp.j.1042.2014.01889

[ref21] Jiang Y. Zhang L. Mao Z. (2015). The influence factors of emotional effects of physical exercise. Stud. Psychol. Behav. 13, 328–333. doi: 10.3969/j.issn.1672-0628.2015.03.007

[ref22] Kai Y. Nagamatsu T. Kitabatake Y. Sensui H. (2016). Effects of stretching on menopausal and depressive symptoms in middle-aged women: a randomized controlled trial. Menopause 23, 827–832. doi: 10.1097/gme.0000000000000651, 27300113 PMC4961267

[ref23] Kirschbaum C. Pirke K. M. Hellhammer D. H. (1993). The ‘Trier social stress test’ – a tool for investigating psychobiological stress responses in a laboratory setting. Neuropsychobiology 28, 76–81. doi: 10.1159/000119004, 8255414

[ref24] Lam L. C. W. Riba M. (2016). Physical Exercise Interventions for Mental Health. Cambridge: Cambridge University Press.

[ref25] Ligeza T. S. Nowak I. Maciejczyk M. Szygula Z. Wyczesany M. (2021). Acute aerobic exercise enhances cortical connectivity between structures involved in shaping mood and improves self-reported mood: an EEG effective-connectivity study in young male adults. Int. J. Psychophysiol. 162, 22–33. doi: 10.1016/j.ijpsycho.2021.01.016, 33508334

[ref26] Meyer J. D. Koltyn K. F. Stegner A. J. Kim J. S. Cook D. B. (2016). Influence of exercise intensity for improving depressed mood in depression: a dose-response study. Behav. Ther. 47, 527–537. doi: 10.1016/j.beth.2016.04.003, 27423168

[ref27] Meyer J. D. Murray T. A. Brower C. S. Cruz-Maldonado G. A. Perez M. L. Ellingson L. D. . (2022). Magnitude, timing and duration of mood state and cognitive effects of acute moderate exercise in major depressive disorder. Psychol. Sport Exerc. 61:102172. doi: 10.1016/j.psychsport.2022.102172

[ref28] Miczek K. A. (2010). “Social stress,” in Encyclopedia of Psychopharmacology, ed. Stolerman I. P. (Berlin: Springer).

[ref29] National Academy of Sports Medicine (2021). NASM Essentials of Personal Fitness Training. Burlington: Jones & Bartlett Learning.

[ref30] Noakes T. D. (2000). Physiological models to understand exercise fatigue and the adaptations that predict or enhance athletic performance: physiological models to study exercise. Scand. J. Med. Sci. Sports 10, 123–145. doi: 10.1034/j.1600-0838.2000.010003123.x 10843507

[ref31] Noetel M. Sanders T. Gallardo-Gómez D. Taylor P. Del Pozo Cruz B. van den Hoek D. . (2024). Effect of exercise for depression: systematic review and network meta-analysis of randomized controlled trials. BMJ 384:e075847. doi: 10.1136/bmj-2023-075847 38355154 PMC10870815

[ref33] Parker H. W. Abreu A. M. Sullivan M. C. Vadiveloo M. K. (2022). Allostatic load and mortality: a systematic review and meta-analysis. Am. J. Prev. Med. 63, 131–140. doi: 10.1016/j.amepre.2022.02.003, 35393143

[ref34] Penedo F. J. Dahn J. R. (2005). Exercise and well-being: a review of mental and physical health benefits associated with physical activity. Curr. Opin. Psychiatry 18, 189–193. doi: 10.1097/00001504-200503000-00013, 16639173

[ref35] Shiota M. N. Kalat J. K. (2017). Emotion. Oxford: Oxford University Press.

[ref36] Sudo M. Ando S. (2020). Effects of acute stretching on cognitive function and mood states of physically inactive young adults. Percept. Mot. Skills 127, 142–153. doi: 10.1177/0031512519888304, 31744384

[ref37] Yang J. Hou Y. Yang Y. Zhang Q. (2011). Impact of trier social stress test (TSST) on salivary cortisol secretion. Acta Psychol. Sin. 43, 403–409. doi: 10.3724/SP.J.1041.2011.00403

[ref38] Yu X. Zhao G. Wang D. Wang S. Li R. Li A. . (2022). A specific circuit in the midbrain detects stress and induces restorative sleep. Science 377, 63–72. doi: 10.1126/science.abn0853, 35771921 PMC7612951

[ref39] Zhu B. (1995). Brief introduction of POMS and Chinese norm. J. Tianjin Inst. Phys. Educ. 1, 35–37.

